# Genetic Variants of *IDE-KIF11-HHEX* at 10q23.33 Associated with Type 2 Diabetes Risk: A Fine-Mapping Study in Chinese Population

**DOI:** 10.1371/journal.pone.0035060

**Published:** 2012-04-10

**Authors:** Yun Qian, Feng Lu, Meihua Dong, Yudi Lin, Huizhang Li, Jian Chen, Chong Shen, Guangfu Jin, Zhibin Hu, Hongbing Shen

**Affiliations:** 1 Department of Epidemiology and Biostatistics, School of Public Health, Nanjing Medical University, Nanjing, China; 2 Department of Chronic Non-communicable Disease Control, Wuxi Center for Disease Control and Prevention, Wuxi, China; Tulane School of Public Health and Tropical Medicine, United States Minor Outlying Islands

## Abstract

**Background:**

Genome-wide association studies (GWAS) in populations of European ancestry have mapped a type 2 diabetes susceptibility region to chromosome 10q23.33 containing *IDE*, *KIF11* and *HHEX* genes (*IDE-KIF11-HHEX*), which has also been replicated in Chinese populations. However, the functional relevance for genetic variants at this locus is still unclear. It is critical to systematically assess the relationship of genetic variants in this region with the risk of type 2 diabetes.

**Methodology/Principal Findings:**

A fine-mapping study was conducted by genotyping fourteen tagging single-nucleotide polymorphisms (SNPs) in a 290-kb linkage disequilibrium (LD) region using a two-stage case-control study of type 2 diabetes in a Chinese Han population. Suggestive associations (*P*<0.05) observed from 1,200 cases and 1,200 controls in the first stage were further replicated in 1,725 cases and 2,081 controls in the second stage. Seven tagging SNPs were consistently associated with type 2 diabetes in both stages (*P*<0.05), with combined odds ratios (ORs) ranging from 1.14 to 1.33 in the combined analysis. The most significant locus was rs7923837 [OR = 1.33, 95% confidence interval (CI): 1.21–1.47] at the 3′-flanking region of *HHEX* gene. SNP rs1111875 was found to be another partially independent locus (OR = 1.23, 95% CI: 1.13–1.35) in this region that was associated with type 2 diabetes risk. A cumulative effect of rs7923837 and rs1111875 was observed with individuals carrying 1, 2, and 3 or 4 risk alleles having a 1.27, 1.44, and 1.73-fold increased risk, respectively, for type 2 diabetes (*P* for trend = 4.1E-10).

**Conclusions/Significance:**

Our results confirm that genetic variants of the *IDE-KIF11-HHEX* region at 10q23.33 contribute to type 2 diabetes susceptibility and suggest that rs7923837 may represent the strongest signal related to type 2 diabetes risk in the Chinese Han population.

## Introduction

Type 2 diabetes is one of major public health problems around the world with an affected number of 240 million in 2007, with an expected rapid increase to 380 million by 2025 [1]. In China, about 92.4 million adults (≥20 years old) are affected with diabetes while about 148.2 million adults are in a status of prediabetes [2]. Type 2 diabetes is a serious metabolic disorder, characterized by insulin resistance and relative insulin deficiency, which results from both genetic and environmental factors. The increasing prevalence of type 2 diabetes is largely attributed to environmental factors acting on genetically susceptible individuals. Therefore, it is important to understand the susceptibility genes for type 2 diabetes to facilitate risk assessment, primary prevention, early detection and treatment.

Genome-wide association study (GWAS) has made spectacular progress in identifying susceptibility genes involved in type 2 diabetes. In 2007, the first GWAS of type 2 diabetes conducted in a French case-control cohort identified a type 2 diabetes related locus on chromosome 10q23.33, which is located in a gene cluster including the insulin-degrading enzyme (*IDE*), the kinesin-interacting factor 11 (*KIF11*), and the hematopoietically expressed homeobox (*HHEX*) [3]. This association has been consistently replicated in the subsequent studies [4]–[9], including several studies in Chinese populations [10]–[17]. *HHEX* encodes a transcription factor with a key role for pancreatic development [18]. Reduction of *IDE* activity by a pharmacological inhibitor increases islet amyloid polypeptide (amylin) accumulation and amylin-mediated cytotoxicity in cultured β-cells [19], whereas *IDE* ablation causes glucose intolerance in knockout mice [20]. Therefore, both *IDE* and *HHEX* genes are strong candidates as susceptibility genes modulating the pathogenesis of type 2 diabetes.

The *IDE*-*KIF11*-*HHEX* locus spans a 290 kb region in linkage equilibrium (LD) (Figure S1). Two variants at the 3′-flanking region of the *HHEX* gene, rs7923837 and rs1111875, have been associated with type 2 diabetes risk as lead single-nucleotide polymorphisms (SNPs) in original GWAS in populations of European ancestry. These two SNPs do not reside within the coding or putative regulatory regions of any known genes. It is still an open topic which variant(s) at *IDE*-*KIF11*-*HHEX* locus is (are) causal, especially in different populations. Herein, with the aim to systematically evaluate the relationship between genetic variants at 10q23.33 and type 2 diabetes risk and provide evidence for causal variant(s) in this gene region, we conducted a fine-mapping study including 14 tagging SNPs at 10q23.33 in a large, two-stage case-control study with a total of 2,925 cases and 3,281 controls in a Chinese population.

## Methods

### Ethics statement

This study was approved by the Ethical Committee of Nanjing Medical University. Written informed consent was obtained from each participant before investigation.

### Study population

In this study, we performed a two-stage case-control study. The first-stage (discovery-phase) fine-mapping analysis was designed to discover the suggestive variants associated with type 2 diabetes in a Chinese population consisting of 1,200 cases and 1,200 controls from a community based cohort study of type 2 diabetes in Wuxi, a city of southern Jiangsu Province, China. In this study, eligible subjects aged over 30 years old were enrolled in 2007 and the baseline information, including demographic, disease history, family history of diabetes was obtained and a detailed clinical examination was conducted. Anthropometric variables including height, weight, waist and hip circumference and blood pressure were measured. Body mass index (BMI) was calculated as weight (in kilograms) divided by the square of height (in meters). Ten hours overnight fasting blood samples were drawn for measurements of fasting blood glucose (FBG) and lipids in all subjects. Glucose concentration and lipids were measured on an OLYMPUS (C2734-Au640) automatic analyzer in the central laboratory of Wuxi Center for Disease Control and Prevention, which was authorized to perform laboratory tests according to the international quality standard ISO/IEC 17025. At baseline, 1,200 subjects who had a history of type 2 diabetes and were on medical treatment for type 2 diabetes were selected as type 2 diabetes cases. The control subjects were selected from those without history of diabetes, hypertension, coronary heart disease, stroke, cancer and with a FBG<5.6 mmol/l at both baseline and follow-up in 2009 and were frequency-matched to the cases on sex (n = 1,200).

The second-stage (replication-phase) was to confirm the results observed in the first-stage, and consisted of 1,725 cases and 2,081 controls derived from a community-based cross-sectional survey on chronic non-communicable diseases in 2009 in Nantong, a city in middle Jiangsu Province, China. All study participants aged over 30 years were interviewed face-to-face by trained interviewers using a pre-tested questionnaire including demographic, behaviors, disease history, and family history of diabetes. Anthropometric parameters and blood pressure were measured. Over 10-hour fasting blood samples were collected for measurement of blood glucose and lipids. Subjects were considered to have type 2 diabetes if they had a history of type 2 diabetes or if their fasting glucose was 7.0 mmol/l or higher. Eventually, 1,725 subjects were recruited as cases. Meanwhile, we selected 2,081 subjects without history of diabetes, hypertension, coronary heart disease, stroke, cancer and with FBG<5.6 mmol/l as controls from the same population and also frequency-matched to the cases on sex.

Individuals with the following conditions were excluded from the study at both stages: malnutrition (BMI<18.5 kg/m^2^); physical disabilities or psychological disorder; obesity caused by other diseases (such as islet cell tumor, Cushing's syndrome, polycystic ovary syndrome, hypogonadism, hypothyroidism) or by medication (such as glucocorticoid, oral contraceptive); cancer; current diagnosis of any communicable disease. All subjects in this study were unrelated ethnic Han Chinese. All blood specimens were stored at the Key Molecular Epidemiology Laboratory, Department of Epidemiology and Biostatistics, School of Public Health, Nanjing Medical University. Genomic DNA was extracted from a leukocyte pellet by proteinase K digestion and was followed by phenol-chloroform extraction and ethanol precipitation.

### SNPs selection

We selected both potentially functional SNPs and haplotype-tagging SNPs (htSNP) in the *IDE-KIF11-HHEX* locus at 10q23.33 (chr10:94199856–94489557). Firstly, to infer potentially functional SNPs, we searched common (MAF≥0.10) SNPs located in the 5′-flanking region, 5′untranslated region (5′UTR), exon, and 3′UTR of *IDE*, *KIF11* and *HHEX* genes using NCBI dbSNP database and 8 SNPs (rs7078243, rs1044153, rs11595187, rs11187083, rs2297743, rs3758505, rs7099761, rs4646954) were found. As there isn't any information on rs1044153, rs7099761, rs4646954 in HapMap database, we conducted LD analysis on the other 5 SNPs (rs7078243,rs11595187, rs11187083, rs2297743, rs3758505). Among them, 3 SNPs (rs2297743, rs7078243, rs11187083) were finally selected for this study, because the other 2 SNPs (rs3758505 and rs11595187) are in high LD with rs2297743 (r^2^ = 0.97 and r^2^ = 0.96, respectively). Secondly, we directly included three SNPs (rs1111875, rs5015480, rs7923837) which were significantly associated with type 2 diabetes risk in previous GWAS.

Then we used the block-based tagging strategy to find tagging SNPs using Haploview 4.2 software according to the HapMap database (http://www.hapmap.org/, phaseII Nov08, on NCBI B36 assembly, dbSNP b126; population: Chinese Han population (CHB) and Japanese population (JPT)); MAF≥0.10, Hardy-Weinberg equilibrium *P*≥0.05 and call rate ≥95%) on the basis of pairwise LD r^2^ threshold of 0.8. After forced including the above 6 SNPs (rs2297743, rs7078243, rs11187083, rs1111875, rs5015480, rs7923837) as tagging SNPs into Haploview software, the additional 13 SNPs (rs11187096, rs4646957, rs7911264, rs2488073, rs947591, rs10882084, rs11187146, rs7910605, rs2488075, rs7918084, rs11187094, rs6583826, rs4933236) that met the above criteria in this *IDE-KIF11-HHEX* region were selected. As a result, a total of 19 SNPs were selected for genotyping in the discovery stage (Figure S1).

### Genotype determination

In the discovery stage, genotyping was performed using the TaqMan OpenArray Genotyping System (Life Technologies, Carlsbad, USA), a medium-throughput genotyping platform. DNA samples with standardized concentration were loaded and amplified on 48-sample arrays following the manufacturer's protocol. For quality control, the equal amounts of cases and controls and two no template controls (NTCs) were simultaneously detected in each chip. Four SNPs (rs2297743, rs11187096, rs2488073 and rs7918084) were excluded because of deficiencies in probes design in the chip and the SNP rs6583826 was excluded from analysis because of low call rate (<80.0%). The overall call rate for the remaining 14 SNPs was 98.9%, with a call rate >97.0% for each locus.

In the replication stage with 1,725 type 2 diabetes cases and 2,081 controls, iPLEX Sequenom MassARRAY platform (Sequenom, Inc) was used to genotype the 11 SNPs that were significant in the discovery stage. Genotyping was conducted blindly and two NTCs in each 384-sampleplate were used for quality control. The overall call rate of this stage was 99.4%, with a call rate >99% individually.

Except for rs7078243 in the discovery stage (*P* = 0.048) and rs11187094 in the replication stage (*P* = 0.002), the genotype distribution of other SNPs were all in Hardy-Weinberg equilibrium.

### Statistical analyses

χ^2^ or Student's *t* tests were used to examine the differences in the distributions of characteristics between type 2 diabetes cases and controls. Hardy-Weinberg equilibrium was tested using a likelihood ratio test. LD between SNPs was evaluated using Haploview version 4.2. Genotype distributions between cases and controls were compared using logistic regression under the additive genetic model with adjustment for age, sex and BMI as confounding factors. The combined effect of multiple SNPs on the risk of type 2 diabetes was determined by logistic regression after categorizing the participants into groups according to the number of the risk alleles carried. Individuals with no risk alleles served as the reference group. Cochran's χ^2^ -based Q-statistic was performed to assess heterogeneity in subgroups. Meta-analysis of 9 studies [10]–[17 and this study] in Chinese populations was conducted to estimate the pooled effect size. Heterogeneity of the 9 studies was assessed with the Cochran's χ^2^ -based Q-statistic. The random-effects model was adopted when heterogeneity existed; otherwise the fixed-effects model was appropriate. Combined ORs were calculated using the Mantel-Haenszel (fixed-effects) and DerSimonian and Laird (random-effects) tests [21], [22]. The significant *P* value of overall ORs was determined using the Z-test. All analyses were performed using the PLINK 1.07 and Stata software (version 11.1; StataCorp LP, College Station, Texas).

## Results

The characteristics of the study populations are presented in Table 1. No significant differences were observed in the distributions of sex in both stages and the combined analysis (*P*>0.05). Type 2 diabetes cases were in a higher age than controls (*P*<0.01) and had significantly higher levels of BMI, FBG, triglycerides (TG), total cholesterol (TC) and significantly lower level of high density lipoprotein-cholesterol (HDL-C) as compared with controls in both stages and combined all (*P*<0.0001).

**Table 1:** Characteristics of the study populations.

Variables	Stage I			Stage II			Combined all		
	Case	Control	*P*	Cases	Controls	*P*	Cases	Controls	*P*
n (% male)	1200 (39.8)	1200 (39.8)	0.980	1725 (35.9)	2081 (36.3)	0.805	2925 (37.5)	3281 (37.6)	0.953
Age (years)	57.43±9.77	56.43±8.02	0.006	58.77±10.31	56.66±10.81	1.1E-9	58.21±10.11	56.57±9.88	1.2E-10
BMI (kg/m^2^)^a^	24.92±3.42	22.64±2.87	6.6E-66	25.13±3.55	21.82±2.44	7.9E-220	25.05±3.50	22.12±2.63	1.2E-275
FBG (mmol/l)^b^	8.98±3.52	4.51±0.47	4.4E-306	9.07±3.30	4.53±0.56	<0.001^f^	9.03±3.52	4.52±0.53	<0.001^f^
TG (mmol/l)^c^	2.55±2.46	1.32±0.45	5.2E-62	2.58±2.48	0.96±0.39	9.7E-170	2.57±2.47	1.09±0.45	6.4E-227
TC (mmol/l^d^	5.39±1.62	4.64±0.71	1.2E-46	4.70±1.23	4.26±0.82	2.0E-38	4.98±1.44	4.39±0.80	3.7E-85
HDL-C (mmol/l)^e^	1.37±0.34	1.53±0.31	7.5E-30	1.51±0.55	1.68±0.40	3.6E-26	1.46±0.48	1.62±0.38	2.0E-51

Data are means ± SD or numbers (percentage).

a body mass index (BMI),

b fasting blood glucose (FBG),

c triglycerides (TG),

d total cholesterol (TC),

e high density lipoprotein-cholesterol (HDL-C),

f *P* value is too small to be shown using Stata software.

Among the 14 SNPs analyzed in the first stage (Table 2), 11 SNPs were associated with type 2 diabetes risk with a *P* value less than 0.05, including rs4646957, rs7910605, rs11187094, rs7078243, rs7911264, rs1111875, rs5015480, rs11187146, rs7923837, rs2488075, and rs947591. The 11 suggestive SNPs were further to be tested in the second stage with additional 1,725 type 2 diabetes cases and 2,081 controls. As shown in Table 2, there were 7 SNPs (rs4646957, rs1111875, rs5015480, rs11187146, rs7923837, rs2488075, and rs947591) showed similar associations as observed in the first stage and had a *P* value less than 0.05. We further combined the results of two stages for the 7 SNPs that were consistently associated with type 2 diabetes in both stages (*P*<0.05). As presented in Table 3, all of the 7 SNPs showed significant associations with type 2 diabetes risk with effect size (OR) ranging from 1.14 to 1.33. The most significant locus was rs7923837 after the two stages were combined with an OR of 1.33 (95% CI: 1.21–1.47).

**Table 2:** Summary results of associations between 14 tagging SNPs at 10q23.33 and risk of type 2 diabetes in two stages.

			Major	Stage I (N_case/control_ = 1,200/1,200)			Stage II (N_case/control_ = 1,725/2,081)		
SNP	Position	Location	/minor allele	MAF_case/control_ a	OR (95%CI)^b^	*P* b	MAF_case/control_ a	OR (95%CI)^b^	*P* b
rs4646957	94219892	IDE (intron)	C/T	0.256/0.224	1.22 (1.06–1.41)	0.007	0.250/0.231	1.16 (1.02–1.31)	0.023
rs7910605	94229773	IDE (intron)	A/G	0.181/0.160	1.20 (1.02–1.41)	0.033	0.175/0.161	1.12 (0.97–1.29)	0.119
rs10882084	94319093	intergene	A/G	0.106/0.093	1.21 (0.98–1.49)	0.073	–	–	–
rs11187083	94342485	KIF11 (5′ near gene)	A/T	0.111/0.109	1.06 (0.87–1.29)	0.553	–	–	–
rs11187094	94358158	KIF11 (intron)	A/G	0.463/0.418	1.26 (1.11–1.42)	3.0E-4	0.405/0.396	1.05 (0.95–1.17)	0.339
rs7078243	94404243	KIF11 (3′UTR)	C/A	0.202/0.157	1.42 (1.20–1.67)	3.0E-5	0.181/0.168	1.08 (0.94–1.24)	0.297
rs7911264	94426831	intergene	C/T	0.241/0.199	1.34 (1.15–1.56)	1.5E-4	0.224/0.208	1.09 (0.96–1.24)	0.184
rs1111875	94452862	intergene	T/C	0.299/0.251	1.33 (1.16–1.53)	4.8E-5	0.285/0.261	1.15 (1.02–1.30)	0.019
rs5015480	94455539	intergene	T/C	0.186/0.160	1.27 (1.08–1.49)	0.005	0.184/0.163	1.23 (1.07–1.42)	0.004
rs11187146	94468335	intergene	C/G	0.358/0.331	1.14 (1.01–1.30)	0.042	0.350/0.325	1.14 (1.02–1.28)	0.018
rs7923837	94471897	intergene	A/G	0.235/0.194	1.33 (1.15–1.54)	1.7E-4	0.235/0.199	1.34 (1.18–1.52)	1.0E-5
rs2488075	94480154	intergene	T/C	0.169/0.139	1.32 (1.12–1.56)	0.001	0.171/0.140	1.34 (1.15–1.55)	1.1E-4
rs947591	94485733	intergene	C/A	0.185/0.158	1.28 (1.09–1.50)	0.003	0.187/0.162	1.21 (1.06–1.40)	0.007
rs4933236	94488416	intergene	C/T	0.340/0.322	1.09 (0.95–1.23)	0.214	–	–	–

a Minor allele frequency (MAF) in cases and controls.

b Odds ratio (OR) and *P* value derived from logistic regression with a adjustment for age, sex and BMI assuming a additive genetic model.

**Table 3:** Combined results for 7 SNPs consistently associated with type 2 diabetes risk in two stages.

SNP	Case	Control	Heterozygote *vs.* major homozygote^b^		Minor homozygote *vs.* major homozygote^b^		Additive model^b^	
	(n = 2925)^a^	(n = 3281)^a^	OR (95%CI)	*P*	OR (95%CI)	*P*	OR (95%CI)	*P*
rs4646957	5.86/38.76/55.38	5.39/34.92/59.69	1.26 (1.12–1.42)	1.3E-4	1.22 (0.95–1.56)	0.115	1.18 (1.08–1.30)	3.8E-4
rs1111875	7.88/42.31/49.81	6.80/37.92/55.28	1.31 (1.17–1.48)	5.7E-6	1.36 (1.09–1.69)	0.007	1.23 (1.13–1.35)	5.4E-6
rs5015480	2.83/31.31/65.86	2.61/27.17/70.22	1.33 (1.17–1.50)	8.7E-6	1.20 (0.85–1.70)	0.303	1.25 (1.12–1.39)	4.4E-5
rs11187146	12.38/45.92/41.69	11.04/43.44/45.52	1.18 (1.05–1.33)	0.007	1.27 (1.06–1.53)	0.011	1.14 (1.05–1.24)	0.002
rs7923837	5.38/36.22/58.40	3.90/31.71/64.39	1.37 (1.21–1.54)	3.8E-7	1.66 (1.27–2.18)	2.6E-4	1.33 (1.21–1.47)	6.8E-9
rs2488075	2.69/28.66/68.65	1.90/24.13/73.97	1.36 (1.20–1.55)	2.2E-6	1.60 (1.09–2.33)	0.016	1.33 (1.19–1.49)	3.7E-7
rs947591	3.24/30.80/65.96	2.39/27.37/70.24	1.25 (1.11–1.42)	3.8E-4	1.53 (1.08–2.15)	0.016	1.25 (1.12–1.39)	4.3E-5

a Frequency of minor homozygote/heterozygote/major homozygote.

b Adjusting for age, sex and BMI.

As shown in Figure S2, a moderate LD (r^2^: 0.19–0.64) is indicated between the most significant SNP rs7923837 and the other 6 SNPs (i.e. rs4646957, rs1111875, rs5015480, rs11187146, rs2488075 and rs947591) that were consistently associated with the risk of type 2 diabetes in the present study. After being conditioned by rs7923837, rs1111875 was the only SNP still with a *P* value<0.05 (Table 4), suggesting that the effect of rs1111875 could not be fully explained by rs7923837. In contrast, the SNP rs7923837 remained significant after being conditioned by any of the other SNPs (Table 4). We then combined rs1111875 and rs7923837 genotypes to test their joint effects on type 2 diabetes risk. A significant increased risk of type 2 diabetes was detected as the number of risk alleles increased (*P* for trend = 4.1E-10, Table 5). Compared to those without carrying any risk allele, individuals carrying one, two, and three or four risk alleles had a 1.27, 1.44 and 1.73-fold increased risk for developing type 2 diabetes, respectively, while individuals carrying one or more risk alleles had a 1.39-fold increased risk for type 2 diabetes.

**Table 4:** Pairwise conditional analysis for 7 SNPs at 10q23.33 consistently associated with type 2 diabetes risks in additive genetic model.

SNP^a^	rs4646957	rs1111875	rs5015480	rs11187146	rs7923837	rs2488075	rs947591
rs4646957	-	0.325	0.014	0.004	0.232	0.013	0.004
rs1111875	0.003	-	0.029	9.2E-5	0.032	9.3E-4	1.5E-4
rs5015480	0.002	0.249	-	0.002	0.689	0.031	0.004
rs11187146	0.023	0.026	0.174	-	0.106	0.863	0.355
rs7923837	2.9E-6	2.2E-5	5.8E-5	2.6E-7	-	0.004	4.1E-5
rs2488075	8.6E-6	2.8E-5	1.7E-4	2.8E-5	0.462	-	0.003
rs947591	3.2E-4	5.4E-4	0.003	0.006	0.846	0.627	-

a *P* value shown in each cell represents the SNP in the row adjusting for age, sex, BMI and the SNP in the corresponding column. For instance, *P* value of 0.325 for rs4646957 was adjusted for age, sex, BMI and rs1111875.

**Table 5:** Combined effects of rs7923837 and rs1111875 on type 2 diabetes risk.

Risk allele number^a^	Cases	Controls	OR(95%CI)^a^	*P* b
0	1129 (39.12)	1480 (45.51)	1	
1	810 (28.06)	873 (26.84)	1.27 (1.10–1.46)	7.5E-4
2	681 (23.60)	675 (20.76)	1.44 (1.24–1.67)	1.1E-6
3–4	266 (9.22)	224(6.89)	1.73 (1.39–2.15)	8.2E-7
*P* for trend^a^ = 4.1E-10				
0	1129 (39.12)	1480 (45.51)	1	
1–4	1757 (60.88)	1772 (54.49)	1.39 (1.24–1.56)	1.5E-8

a rs7923837-G and rs1111875-C were assumed as risk alleles.

b Adjusted by age, sex and BMI.

Stratification analyses using pooled case-control sets in additive genetic model showed that the associations between the two SNPs (rs1111875 and rs7923837) and type 2 diabetes risk were significant in subgroups stratified by age, sex, BMI, or stage, with ORs ranging from 1.15 to 1.44, and the associations had no significant difference between the subgroups (*P*>0.05 for heterogeneity test, Table S1).

To estimate the pooled effect size of *IDE-KIF11-HHEX* variants on type 2 diabetes risk, we conducted a meta-analysis of the three reported SNPs (rs1111875, rs5015480 and rs7923837) in a total of 14,141 cases and 15,725 controls for rs1111875, 9,208 cases and 11,363 controls for rs5015480, and 8,184 cases and 10,358 controls for rs7923837, respectively, in Chinese populations [10]–[17 and this study]. There was no heterogeneity of ORs across studies of rs1111875 and rs5015480, but there lied heterogeneity among studies of rs7923837 (*P* = 0.044). The pooled ORs for type 2 diabetes were significant for rs1111875 (pooled OR = 1.16, 95% CI = 1.11–1.20, *P*<0.0001), rs5015480 (pooled OR = 1.18, 95% CI = 1.11–1.25, *P*<0.0001) in the fixed-effects model, and for rs7923837 (pooled OR = 1.19, 95% CI = 1.08–1.30, *P*<0.0001) in the random-effects model (Figure S3).

## Discussion

The current study represents the first fine-mapping study with an effort to comprehensively investigate the relationship between *IDE-KIF11-HHEX* locus and type 2 diabetes risk in this Chinese population. This is also the largest study in terms of sample size in a Chinese population to date. We confirmed the associations of genetic variants at *IDE-KIF11-HHEX* gene region with risk of type 2 diabetes in Chinese Han population. The tagging SNP rs7923837 was found to be the variant with the strongest effect in our population, whereas rs1111875 showed an independent effect on type 2 diabetes that could not be fully interpreted by rs7923837. These findings provide new insights into the genetic variants at *IDE-KIF11-HHEX* and susceptibility of type 2 diabetes, and allow for a more stable estimation of effect size on this locus facilitating genetic risk prediction in the future.

Since Sladek *et al* firstly reported the association of genetic variants at *IDE-KIF11-HHEX* with type 2 diabetes risk in 2007 [3], several studies have replicated the significant association in Chinese population [10]–[16]. Among those studies, the SNP rs1111875 was included in six studies and the reported ORs ranged from 1.09 to 1.23 [10], [11], [13]–[16]. Three of them including rs7923837 all reported a higher effect size with ORs of 1.20–1.45 [10], [13], [16] in Chinese population, which was also found in Japanese [23] and European ancestry [24] populations. In the meta-analysis of 22 studies dealing with the relationship between the *HHEX* polymorphism and type 2 diabetes in different ethnicities including Asian, Caucasian, Indian and African American, the summary per-allele OR for type 2 diabetes of the rs1111875 and rs7923837 polymorphism was 1.20 (95% CI: 1.16–1.24), 1.26 (95% CI: 1.19–1.33) in Asians, and 1.16 (95% CI: 1.10–1.23), 1.22 (95% CI: 1.15–1.29) in Europeans, respectively [24]. In addition, SNP rs5015480 was also investigated in 4 studies [10], [12], [13], [16], with the ORs being from 1.08 to 1.32 in Chinese population. In our meat-analysis, the pooled ORs for type 2 diabetes of the rs1111875, rs5015480 and rs7923837 were 1.16 (95% CI: 1.11–1.20), 1.18 (95% CI: 1.11–1.25) and 1.19 (95% CI: 1.08–1.30), respectively. Our findings in this study were consistent with these results and confirmed that *IDE-KIF11-HHEX* locus was one of the susceptibility regions of type 2 diabetes and rs7923837 represented the strongest signal in this region.

We observed a partially independent effect for rs7923837 and rs1111875 on type 2 diabetes in our population. These two loci were in modest LD in our population (r^2^ = 0.20), and similar results were also showed in other Eastern Asian populations [10], [23], but a moderate LD was indicated in 1000 Genomes data for population of European descent (r^2^ = 0.687). The difference in terms of genotype frequency was also evident between Chinese (0.194 and 0.251 for rs7923837-G allele and rs1111875-C allele, respectively) and European ancestry populations (0.592 and 0.658 for rs7923837-G allele and rs1111875-C allele, respectively). These points suggest a marked genetic difference between ethnicities. Importantly, a cumulative effect was observed when the genotypes of rs7923837 and rs1111875 were combined to be analyzed with type 2 diabetes risk, which would be important to accurately estimate the risk of type 2 diabetes using genetic markers in the future.

In this fine-mapping study, we identified the most significant signal of rs7923837 and additional independent signal of rs1111875, both of which are located at the telomeric end of a 290-kb LD block on chromosome 10. As shown in Figure S1, these two SNPs are both in the 3′-flanking region of *HHEX* gene. The *HHEX* gene encodes a transcription factor that is involved in Wnt signaling, a fundamental pathway for cell growth and development [25], and has been shown to regulate β-cell development and/or function through the activation of hepatocyte nuclear factor 1α [26]. Recently, Pivovarova *et al.* found that rs7923837 and rs1111875 were associated with altered capacity of β-cell secretion and proposed that genetic variants at *IDE-KIF11-HHEX* locus might mediate the type 2 diabetes risk by modulating β-cell secretary capacity and β-cell mass [27]. Several studies also reported associations between rs1111875 and rs7923837 and fasting insulin secretion, insulin sensitivity and insulin secretion response following a glucose load [28]–[32]. Therefore, *HHEX* was suggested as the most likely causal candidate gene at 10q23.33 for type 2 diabetes [33]. Nevertheless, *IDE* is also another strong candidate gene. Reduction of *IDE* activity by a pharmacological inhibitor increases islet amyloid polypeptide (amylin) accumulation and amylin-mediated cytotoxicity in cultured β-cells [19] and *IDE* ablation causes glucose intolerance in knockout mice [20]. To now, it is still hard to say which genetic variant(s) in this locus is (are) causal and how they function in the pathogenesis of type 2 diabetes, though our findings provide additional evidence to refine the potential functional variants. Further functional and resequencing studies are warranted to clarify the biological mechanisms of *IDE-KIF11-HHEX* locus on type 2 diabetes and to identify additional variants to narrow down the fine-mapped region.

In summary, our fine-mapping study with a two-stage case-control design and large sample size confirmed the association of *IDE-KIF11-HHEX* locus at 10q23.33 with susceptibility to type 2 diabetes. Our results suggest that tagging SNP rs7923837 may represent the most significant signal in this region in Chinese Han population.

## Supporting Information

Figure S1 **The 290-kb linkage disequilibrium analysis on 10q23.33 and tagging single-nucleotide polymorphisms (chr10:94199856–94489557).** The *IDE-KIF11-HHEX* locus at 10q23.33 (chr10:94199856–94489557) splits into 5 linkage disequilibrium (LD) blocks in Asians (Chinese & Japanese). 19 single-nucleotide polymorphisms (SNPs) (seen at the black arrows) were selected for genotyping in the discovery stage, including 13 haplotype-tagging SNPs (htSNP), chosed with criteria of minor allele frequency (MAF)≥0.10, Hardy-Weinberg equilibrium *P*≥0.05, and call rate ≥95%) on the basis of pairwise LD r^2^ threshold of 0.8, 3 potentially functional SNPs and 3 SNPs previously reported by GWAS of type 2 diabetes.
(DOC)

Figure S2 **Linkage disequilibrium analysis of 7 single-nucleotide polymorphisms consistently associated with type 2 diabetes risks.** Linkage disequilibrium (LD) strength in controls was shown in the diamonds represented by r^2^ values. A moderate LD (r^2^: 0.19–0.64) was indicated between the most significant SNP rs7923837 and the other significant 6 SNPs, with r^2^ value being 0.19 for rs4646957, 0.20 for rs1111875, 0.43 for rs5015480, 0.50 for rs11187146, 0.64 for rs2488075, 0.46 for rs947591 respectively.
(DOC)

Figure S3 **Meta-analysis of 3 single-nucleotide polymorphisms from** ***IDE-KIF11-HHEX*** **locus with type 2 diabetes in Chinese populations.** The pooled odds ratios (ORs) for type 2 diabetes were significant for rs1111875 (pooled OR = 1.16, *P*<0.0001), rs5015480 (pooled OR = 1.18, *P*<0.0001) in the fixed-effects model, and for rs7923837 (pooled OR = 1.19, *P*<0.0001) in the random-effects mode.
(DOC)

Table S1 **Stratification analysis for two independent SNPs (rs7923837 and rs1111875) and risk of type 2 diabetes in additive genetic model.**
(DOC)

## References

[1] International Diabetes Federation (2006). Diabetes Atlas. 3rd ed.

[2] Yang W, Lu J, Weng J, Jia W, Ji L (2010). Prevalence of diabetes among men and women in China.. N Engl J Med.

[3] Sladek R, Rocheleau G, Rung J, Dina C, Shen L (2007). A genome-wide association study identifies novel risk loci for type 2 diabetes.. Nature.

[4] Steinthorsdottir V, Thorleifsson G, Reynisdottir I, Benediktsson R, Jonsdottir T (2007). A variant in CDKAL1 influences insulin response and risk of type 2 diabetes.. Nat Genet.

[5] Saxena R, Voight BF, Diabetes Genetics Initiative of Broad Institute of Harvard and MIT, Lund University, and Novartis Institutes of BioMedical Research (2007). Genome-wide association analysis identifies loci for type 2 diabetes and triglyceride levels.. Science.

[6] Scott LJ, Mohlke KL, Bonnycastle LL, Willer CJ, Li Y (2007). A genome-wide association study of type 2 diabetes in Finns detects multiple susceptibility variants.. Science.

[7] Zeggini E, Weedon MN, Lindgren CM, Frayling TM, Elliott KS (2007). Replication of genome-wide association signals in UK samples reveals risk loci for type 2 diabetes.. Science.

[8] Omori S, Tanaka Y, Takahashi A, Hirose H, Kashiwagi A (2008). Association of CDKAL1, IGF2BP2, CDKN2A/B, HHEX, SLC30A8, and KCNJ11 with susceptibility to type 2 diabetes in a Japanese population.. Diabetes.

[9] Takeuchi F, Serizawa M, Yamamoto K, Fujisawa T, Nakashima E (2009). Confirmation of multiple risk Loci and genetic impacts by a genome-wide association study of type 2 diabetes in the Japanese population.. Diabetes.

[10] Ng MC, Park KS, Oh B, Tam CH, Cho YM (2008). Implication of genetic variants near TCF7L2, SLC30A8, HHEX, CDKAL, CDKN2A/B, IGF2BP2, and FTO in type 2 diabetes and obesity in 6719 Asians.. Diabetes.

[11] Hu C, Zhang R, Wang C, Wang J, Ma X (2009). PPARG, KCNJ11, CDKAL1, CDKN2A-CDKN2B, IDE-KIF11-HHEX, IGF2BP2 and SLC30A8 are associated with type 2 diabetes in a Chinese population.. PLoS One.

[12] Han X, Luo Y, Ren Q, Zhang X, Wang F (2010). Implication of genetic variants near SLC30A8, HHEX, CDKAL1, CDKN2A/B, IGF2BP2, FTO, TCF2, KCNQ1, and WFS1 in type 2 diabetes in a Chinese population.. BMC Med Genet.

[13] Lin Y, Li P, Cai L, Zhang B, Tang X (2010). Association study of genetic variants in eight genes/loci with type 2 diabetes in a Han Chinese population.. BMC Med Genet.

[14] Zhou DZ, Liu Y, Zhang D, Liu SM, Yu L (2010). Variations in/nearby genes coding for JAZF1, TSPAN8/LGR5 and HHEX-IDE and risk of type 2 diabetes in Han Chinese.. J Hum Genet.

[15] Tan JT, Ng DP, Nurbaya S, Ye S, Lim XL (2010). Polymorphisms identified through genome-wide association studies and their associations with type 2 diabetes in Chinese, Malays, and Asian-Indians in Singapore.. J Clin Endocrinol Metab.

[16] Wu Y, Li H, Loos RJ, Yu Z, Ye X (2008). Common variants in CDKAL1, CDKN2A/B, IGF2BP2, SLC30A8, and HHEX/IDE genes are associated with type 2 diabetes and impaired fasting glucose in a Chinese Han population.. Diabetes.

[17] Xu M, Bi Y, Xu Y, Yu B, Huang Y (2010). Combined effects of 19 common variations on type 2 diabetes in Chinese: results from two community-based studies.. PLoS One.

[18] Bort R, Martinez-Barbera JP, Beddington RS, Zaret KS (2004). Hex homeobox gene-dependent tissue positioning is required for organogenesis of the ventral pancreas.. Development.

[19] Bennett RG, Hamel FG, Duckworth WC (2003). An insulin-degrading enzyme inhibitor decreases amylin degradation, increases amylin-induced cytotoxicity, and increases amyloid formation in insulinoma cell cultures.. Diabetes.

[20] Farris W, Mansourian S, Chang Y, Lindsley L, Eckman EA (2003). Insulin-degrading enzyme regulates the levels of insulin, amyloid beta-protein, and the beta-amyloid precursor protein intracellular domain in vivo.. Proc Natl Acad Sci U S A.

[21] Mantel N, Haenszel W (1959). Statistical aspects of the analysis of data from retrospective studies of disease.. J Natl Cancer Inst.

[22] DerSimonian R, Laird N (1986). Meta-analysis in clinical trials.. Control Clin Trials.

[23] Furukawa Y, Shimada T, Furuta H, Matsuno S, Kusuyama A (2007). Polymorphisms in the IDE-KIF11-HHEX gene locus are reproducibly associated with type 2 diabetes in a Japanese population.. J Clin Endocrinol Metab.

[24] Cai Y, Yi J, Ma Y, Fu D (2011). Meta-analysis of the effect of HHEX gene polymorphism on the risk of type 2 diabetes.. Mutagenesis.

[25] Foley AC, Mercola M (2005). Heart induction by Wnt antagonists depends on the homeodomain transcription factor Hex.. Genes Dev.

[26] Tanaka H, Yamamoto T, Ban T, Satoh S, Tanaka T (2005). Hex stimulates the hepatocyte nuclear factor 1alpha-mediated activation of transcription.. Arch Biochem Biophys.

[27] Pivovarova O, Nikiforova VJ, Pfeiffer AF, Rudovich N (2009). The influence of genetic variations in HHEX gene on insulin metabolism in the German MESYBEPO cohort.. Diabetes Metab Res Rev.

[28] Pascoe L, Tura A, Patel SK, Ibrahim IM, Ferrannini E (2007). Common variants of the novel type 2 diabetes genes CDKAL1 and HHEX/IDE are associated with decreased pancreatic beta-cell function.. Diabetes.

[29] Grarup N, Rose CS, Andersson EA, Andersen G, Nielsen AL (2007). Studies of association of variants near the HHEX, CDKN2A/B, and IGF2BP2 genes with type 2 diabetes and impaired insulin release in 10,705 Danish subjects: validation and extension of genome-wide association studies.. Diabetes.

[30] Staiger H, Machicao F, Stefan N, Tschritter O, Thamer C (2007). Polymorphisms within novel risk loci for type 2 diabetes determine beta-cell function.. PLoS One.

[31] Staiger H, Stancáková A, Zilinskaite J, Vänttinen M, Hansen T (2008). A candidate type 2 diabetes polymorphism near the HHEX locus affects acute glucose-stimulated insulin release in European populations: results from the EUGENE2 study.. Diabetes.

[32] Ruchat SM, Elks CE, Loos RJ, Vohl MC, Weisnagel SJ (2009). Association between insulin secretion, insulin sensitivity and type 2 diabetes susceptibility variants identified in genome-wide association studies.. Acta Diabetol.

[33] van Vliet-Ostaptchouk JV, Onland-Moret NC, van Haeften TW, Franke L, Elbers CC (2008). HHEX gene polymorphisms are associated with type 2 diabetes in the Dutch Breda cohort.. Eur J Hum Genet.

