# Supplementary material for: Genetic Variants of IDE-KIF11-HHEX at 10q23.33 Associated with Type 2 Diabetes Risk: A Fine-Mapping Study in Chinese Population
Source: PLoS One. 2012 Apr 10;7(4):e35060. doi: 10.1371/journal.pone.0035060 (PMC3323633; doi:10.1371/journal.pone.0035060)

**Figure S1. The 290-kb linkage disequilibrium analysis on 10q23.33 and tagging single-nucleotide polymorphisms（chr10:94199856-94489557）.**


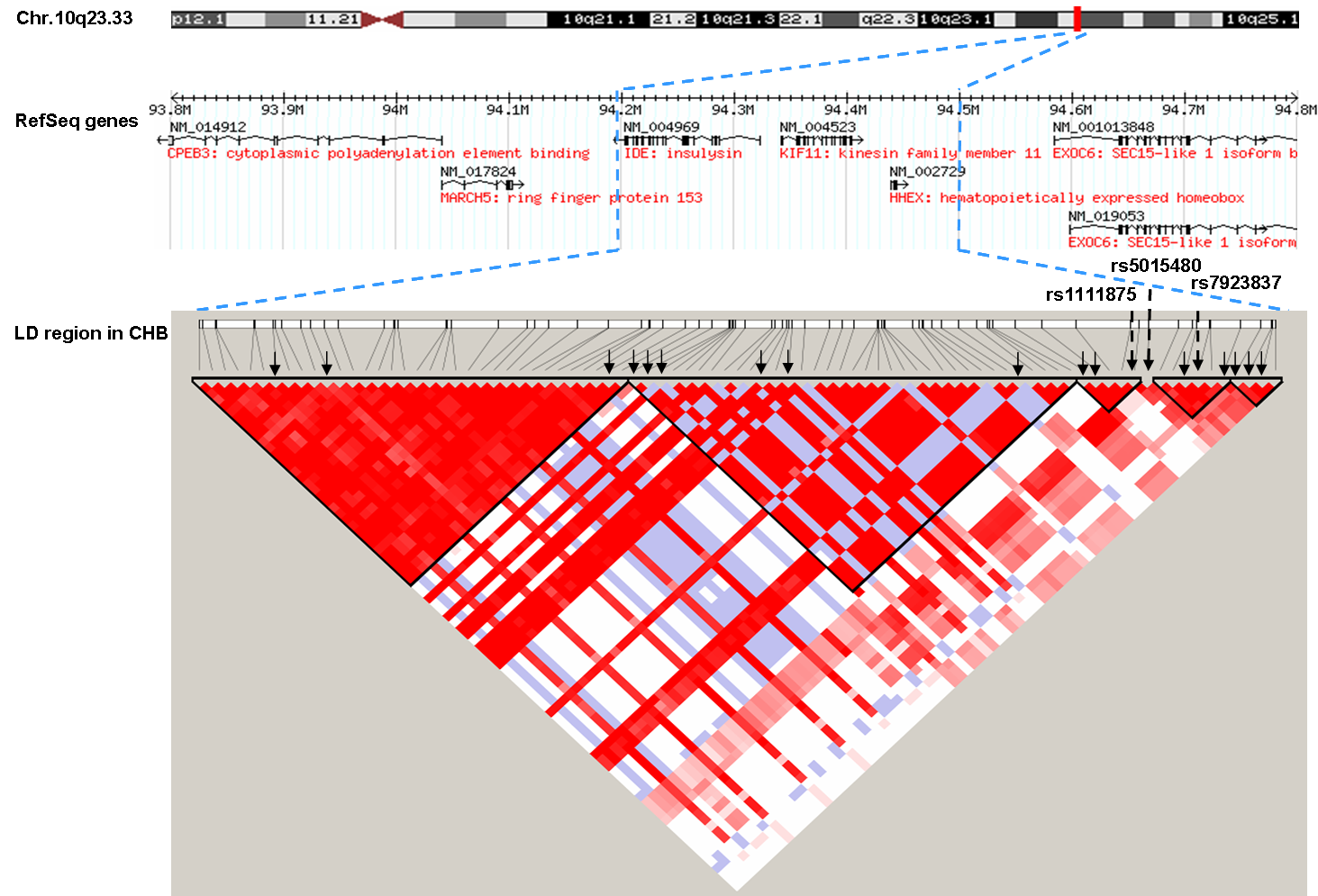

Supplement: Figure S1 — The 290-kb linkage disequilibrium analysis on 10q23.33 and tagging single-nucleotide polymorphisms (chr10:94199856–94489557). The IDE-KIF11-HHEX locus at 10q23.33 (chr10:94199856–94489557) splits into 5 linkage disequilibrium (LD) blocks in Asians (Chinese & Japanese). 19 single-nucleotide polymorphisms (SNPs) (seen at the black arrows) were selected for genotyping in the discovery stage, including 13 haplotype-tagging SNPs (htSNP), chosed with criteria of minor allele frequency (MAF)≥0.10, Hardy-Weinberg equilibrium P≥0.05, and call rate ≥95%) on the basis of pairwise LD r2 threshold of 0.8, 3 potentially functional SNPs and 3 SNPs previously reported by GWAS of type 2 diabetes. (DOC) [file pone.0035060.s001.doc]
