# Supplementary material for: Genetic Variants of IDE-KIF11-HHEX at 10q23.33 Associated with Type 2 Diabetes Risk: A Fine-Mapping Study in Chinese Population
Source: PLoS One. 2012 Apr 10;7(4):e35060. doi: 10.1371/journal.pone.0035060 (PMC3323633; doi:10.1371/journal.pone.0035060)

**Figure S2. Linkage disequilibrium analysis of 7 single-nucleotide polymorphisms consistently associated with type 2 diabetes risks.**


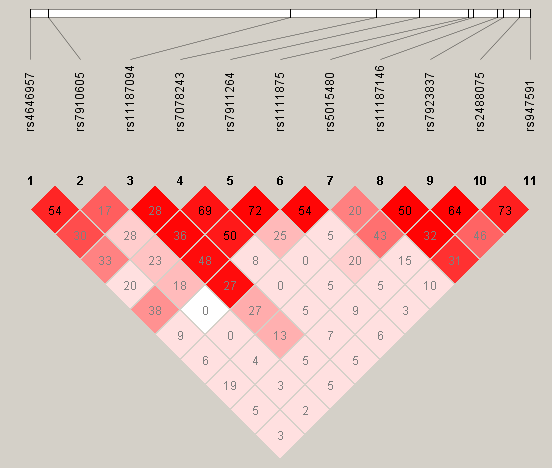

Supplement: Figure S2 — Linkage disequilibrium analysis of 7 single-nucleotide polymorphisms consistently associated with type 2 diabetes risks. Linkage disequilibrium (LD) strength in controls was shown in the diamonds represented by r2 values. A moderate LD (r2: 0.19–0.64) was indicated between the most significant SNP rs7923837 and the other significant 6 SNPs, with r2 value being 0.19 for rs4646957, 0.20 for rs1111875, 0.43 for rs5015480, 0.50 for rs11187146, 0.64 for rs2488075, 0.46 for rs947591 respectively. (DOC) [file pone.0035060.s002.doc]
