# Supplementary material for: Genetic Variants of IDE-KIF11-HHEX at 10q23.33 Associated with Type 2 Diabetes Risk: A Fine-Mapping Study in Chinese Population
Source: PLoS One. 2012 Apr 10;7(4):e35060. doi: 10.1371/journal.pone.0035060 (PMC3323633; doi:10.1371/journal.pone.0035060)
Supplement: Figure S3 — Meta-analysis of 3 single-nucleotide polymorphisms from IDE-KIF11-HHEX locus with type 2 diabetes in Chinese populations. The pooled odds ratios (ORs) for type 2 diabetes were significant for rs1111875 (pooled OR = 1.16, P<0.0001), rs5015480 (pooled OR = 1.18, P<0.0001) in the fixed-effects model, and for rs7923837 (pooled OR = 1.19, P<0.0001) in the random-effects mode. (DOC) [file pone.0035060.s003.doc]

**Figure S3. Meta-analysis of 3 single-nucleotide polymorphisms from *IDE-KIF11-HHEX* locus with type 2 diabetes in Chinese populations.**

rs1111875 and type 2 diabetes

rs5015480 and type 2 diabetes

rs7923837 and type 2 diabetes
