# Supplementary material for: Genetic Variants of IDE-KIF11-HHEX at 10q23.33 Associated with Type 2 Diabetes Risk: A Fine-Mapping Study in Chinese Population
Source: PLoS One. 2012 Apr 10;7(4):e35060. doi: 10.1371/journal.pone.0035060 (PMC3323633; doi:10.1371/journal.pone.0035060)
Supplement: Table S1 — Stratification analysis for two independent SNPs (rs7923837 and rs1111875) and risk of type 2 diabetes in additive genetic model. (DOC) [file pone.0035060.s004.doc]

**Table S1. Stratification analysis for two independent SNPs (rs7923837 and rs1111875) and risk of type 2 diabetes in additive genetic model.**

| **Variables** | **rs7923837** | | | ***P*^c^** | **rs1111875** | | | ***P*^c^** |
| --- | --- | --- | --- | --- | --- | --- | --- | --- |
|  | **Cases^a^** | **Controls^a^** | **OR(95%CI)^b^** |  | **Cases^a^** | **Controls^a^** | **OR(95%CI)^b^** |  |
| Age | | | | | | | | |
| ≤56 | 63/428/631 | 60/470/914 | 1.30(1.12-1.51) | 0.762 | 87/502/533 | 104/550/788 | 1.25(1.09-1.43) | 0.720 |
| >56 | 93/621/1062 | 67/563/1184 | 1.34(1.18-1.52) | | 141/721/908 | 118/688/1017 | 1.21(1.08-1.36) | |
| Sex | | | | | | | | |
| female | 99/672/1042 | 79/678/1277 | 1.28(1.13-1.44) | 0.248 | 145/751/915 | 139/789/1108 | 1.17(1.04-1.31) | 0.133 |
| male | 57/378/651 | 48/355/821 | 1.44(1.23-1.69) | | 83/473/526 | 83/449/697 | 1.35(1.17-1.57) | |
| BMI^d^ | | | | | | | | |
| ＜24 | 61/424/627 | 98/842/1662 | 1.32(1.17-1.49) | 0.880 | 88/491/532 | 181/985/1439 | 1.24(1.11-1.38) | 0.596 |
| ≥24 | 95/620/1049 | 28/191/431 | 1.30(1.11-1.52) | | 139/727/893 | 41/252/361 | 1.18(1.02-1.37) | |
| Stage | | | | | | | | |
| Stage I | 65/430/696 | 47/365/773 | 1.33(1.15-1.55) | 0.940 | 100/510/579 | 77/445/671 | 1.33(1.16-1.53) | 0.121 |
| Stage II | 91/620/997 | 80/668/1325 | 1.34(1.18-1.52) | | 128/714/862 | 145/793/1134 | 1.15(1.02-1.30) | |

^a^ Variant homozygote/heterozygote/wild-type homozygote.

^b^ OR(95%CI) was calculated in logistic regression in additive genetic model, adjusted for age, sex and BMI where appropriate.

^c^ *P* for heterogeneity test..

^d^ BMI of 24 is recommended as the cutoff point for overweight in Chinese.
